# Mangiferin relieves CCl4-induced liver fibrosis in mice

**DOI:** 10.1038/s41598-023-30582-3

**Published:** 2023-03-13

**Authors:** Lijun Zhang, Chuhe Liu, Liufang Yin, Cheng Huang, Shengjie Fan

**Affiliations:** School of Pharmacy, Shanghai University of Traditional Chinese Medicine, Shanghai, 201203 China

**Keywords:** Drug discovery, Health care, Medical research

## Abstract

Hepatic fibrosis is a late stage process of many chronic liver diseases. Blocking the fibrosis process will be beneficial to the treatment and recovery of the diseases. Mangiferin has many pharmacological activities. Recently, it has been reported that mangiferin may relieve tissue fibrosis, including renal, myocardial, pulmonary fibrosis via anti-inflammatory and anti-oxidative effects in animal models. Here, we investigate the effects of mangiferin on CCl4-induced liver fibrosis and the underlying mechanism in mice. Thirty-two male C57BL/6 mice were randomly divided into 4 groups (n = 8 in each group), injected with carbon tetrachloride (10% CCl4) for 8 weeks, and oral administrated with mangiferin (50 mg/kg or 100 mg/kg) from the fifth week. The serum levels of ALT, AST were analyzed to evaluate liver function. H&E, Masson’s trichrome and Sirius red staining were used to assess liver morphology and the degree of liver fibrosis. Quantitative RT-PCR and Western blot were used to assay the gene expression and protein levels. The results showed that mangiferin alleviated the serum levels of AST, ALT, ALP, TBA and TBIL, reduced liver lesions, prevented hepatic parenchymal necrosis, and ameliorated collagen accumulation in the liver of CCl4-treated mice. Meanwhile, mangiferin inhibited the expression of inflammatory genes IL-6 and IL-1β, fibrogenic genes α-SMA, TGF-β and MMP-2 and bile acid metabolism genes ABCB4, ABCB11, SULT2A1 in the liver of CCl4-treated mice. Furthermore, mangiferin reduced collagen accumulation and HSCs activation, inhibited the p-IκB and p-p65 protein levels. Our results suggest that mangiferin could alleviate liver fibrosis in CCl4-treated mice through inhibiting NF-κB signaling, and mango consuming may have beneficial effects to hepatic fibrosis.

## Introduction

Fibrosis is a pathological condition caused by an abnormal process of tissue regeneration in response to chronic injury, which leads to continuous activation of fibroblasts and permanent tissue damage^1,2^. Liver fibrosis is an important global health problem, characterized by the excessive deposition of extracellular matrix (ECM) and the structural disorder of the liver^3^, which eventually leads to the end stage of liver diseases such as liver cirrhosis and hepatocellular carcinoma. There are many causes for liver fibrosis, such as viral hepatitis, alcoholic liver, fatty liver disease and autoimmune diseases and so on. Among the activated fibrous cells, hepatic stellate cells (HSCs) play the most important role in the development of hepatic fibrosis^4^, which could transform into proliferative and contractile myofibroblast-like cells, one of the sources of the accumulation of ECM^5^. In addition, hepatocytes, portal fibroblasts, and bone marrow derived myofibroblasts precursors are the source of ECM^6^. At present, the therapeutic intervention of liver fibrosis involves in preventing the stimulus or harmful cause, inhibiting hepatic inflammation, interfering in the activation of stellate cells and promoting the deterioration of extracellular matrix^7^.

In animals, various causes of fibrogenesis have been studied, but the main hepatic fibrosis model is based on repeated application of carbon tetrachloride (CCl4) over a period of several weeks^8^. CCl4 is a hepatotoxin that causes lobular central hepatic necrosis, proinflammatory and profibrotic cytokine release, and the metabolic activation in the liver^9^, consequently, results in liver fibrosis and even cirrhosis after long-term exposure.

Mangiferin (1, 3, 6, 7-tetrahydroxyxanthone-C2-β-D-glucoside), a naturally occurred C-glucosyl xanthone, is primarily isolated from mango (*Anacardiaceae*), and exists in at least 16 plant families, including *Iridaceae* and *Gentianaceae*^10^. It has been reported that mangiferin has strong antioxidant^11^ and anti-inflammatory^12^ effects and may exert beneficial properties to acute inflammation of the organs such as lung^13^, kidney^14^ and cardiovascular system. Moreover, it has also been reported that mangiferin has a wide range of pharmacological roles, including immunomodulatory^15^, anticancer^16^, antibacterial, antiviral and neuroprotective^17^ effects. The emerging evidence has shown that mangiferin could alleviate organ fibrosis^18^, including renal fibrosis^19,20^, myocardial fibrosis^21^, pulmonary fibrosis^22^ via restraining myofibroblast activation, anti-oxidant and anti-inflammatory functions. Furthermore, Hou et al. found that oral administration of mangiferin to adults did not cause significant side effects, suggesting that there is no toxicity to human^23^. Therefore, mangiferin has been shown as potential candidate for anti-fibrosis agent. In the present study, we investigate whether mangiferin has a therapeutic effect in CCl4-induced hepatic fibrosis in mice.

## Results

### Mangiferin attenuates CCl4 induced liver fibrosis in mice

**Figure 1:**
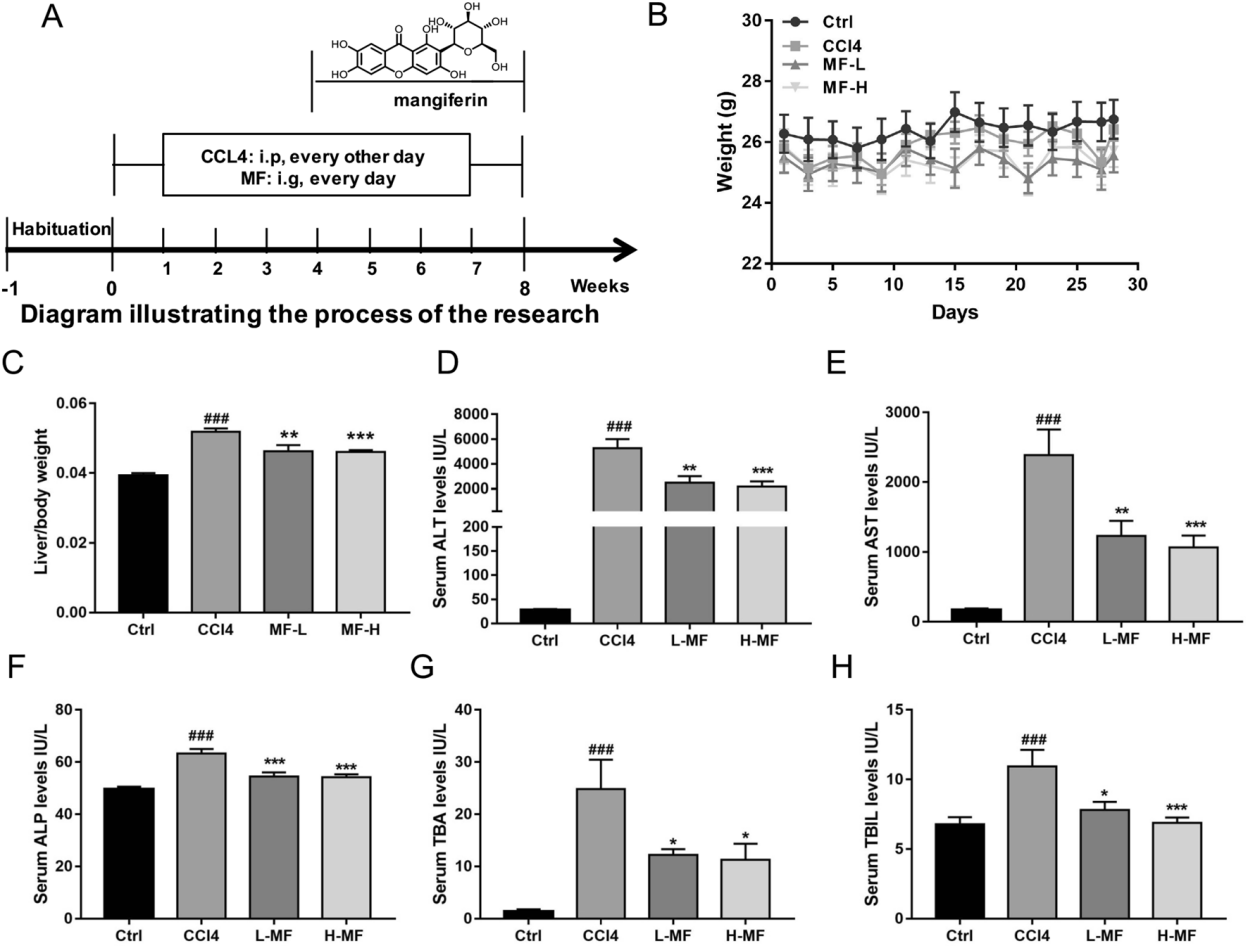
Mangiferin relieves liver injury induced by CCl4. (**A**) The diagram of treatment of mouse model. (**B**) Body weight. (**C**) The liver/body weight. (**D**) Serum ALT level. (**E**) Serum AST level. (**F**) Serum ALP level. (**G**) Serum TBA level. (**H**) Serum TBIL level. The mice were injected intraperitoneally 10% CCl4 (CCl4 was dissolved in olive oil) at dose of 2 mg/kg of body weight for 8 weeks, the control group mice were injected with an equal amount of olive oil. The mangiferin treatment group were injected intraperitoneally 10% CCl4 and orally administrated mangiferin at the dose of 50 mg/kg and 100 mg/kg (0.1 ml/10 g of body weight). Date are presented as means ± S.E.M. (n = 8). ###*p* < 0.001, compared with control group; *p < 0.05, **p < 0.01 and ***p < 0.001, compared with CCl4 group. Ctrl, control group; CCl4, carbon tetrachloride treated group; L-MF, low dose of mangiferin (50 mg/kg); H-MF, high dose of mangiferin (100 mg/kg).

The administration of CCl4 is known to induce toxicity in the liver by producing highly reactive metabolites, resulting in severe damage to liver cells and subsequently developing into fibrosis^24^. In order to determine whether mangiferin could relieve liver injury, mice were intraperitoneally injected with 10% CCl4 for 8 weeks, and mangiferin was given intragastrically every day from the fifth week (Fig. 1A). At the end of the experiment, the liver/body weights were calculated, and the levels of AST, ALT, ALP, TBA and TBIL were measured. We found that mangiferin reduced the liver/body weight rational increased by CCl4, but did not changed body weight (Fig. 1B,C). The levels of serum ALP, ALT and AST, important markers reflecting liver function^25^, were increased in CCl4-treated group compared to those of CCl4-untreated group, indicating the liver function was impaired by the CCl4 administration. Mangiferin treatment significantly reduced the ALT, AST and ALP level both in low and high dose mangiferin treated groups (Fig. 1D–F). The levels of serum TBA and TBIL, representing the accumulation of bile acids in liver fibrosis, were all increased in CCl4-treated group. Mangiferin treatment also significantly attenuated TBA and TBIL content between CCl4-treated group and mangiferin-treated group (Fig. 1G–H). These data suggest that mangiferin could alleviate liver injury and improve liver function caused by the administration of CCl4.

### Mangifein alleviates liver pathological damages and fibrosis scores in CCl4 treated mice

**Figure 2:**
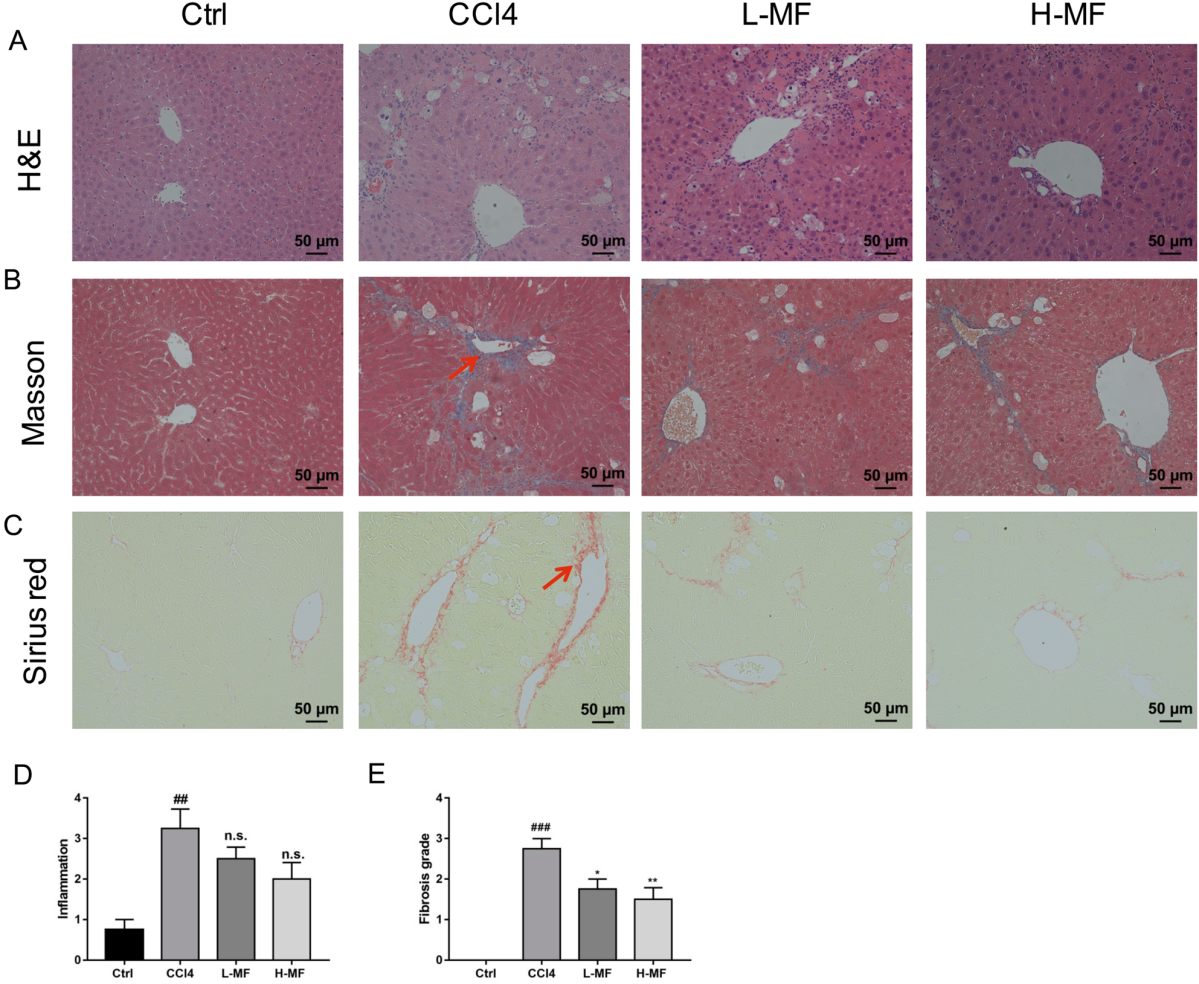
Effects of mangiferin on histology of the liver in CCl4 induced mice. (**A**) H&E staining. (**B**) Masson’s trichrome staining. Red arrows indicated damaged liver tissue and fiber cords. (**C**) Sirius red staining. (**D**) The analysis of inflammation in H&E staining. (**E**) The analysis of fibrosis grade in Masson’s trichrome staining. Date are presented as means ± S.E.M. (n = 8). ##*p* < 0.01 and ###*p* < 0.001, compared with control group. *p < 0.05 and **p < 0.01, compared with CCl4 group. Ctrl, control group; Ctrl, control group; CCl4, carbon tetrachloride treated group; L-MF, low dose of mangiferin (50 mg/kg); H-MF, high dose of mangiferin (100 mg/kg).

Next, we investigated whether mangiferin could relieve liver fibrosis and protect the liver damage from CCl4 toxicity. H&E, Masson’s trichrome and Sirius red staining were used to analyze histology and collagen deposit in the liver section. As shown in Fig. 2A, the livers of control group mice had normal lobular structure with central vein and radial hepatic cord, however, there was necrosis in the center of the hepatic lobules, deposition of lipid droplets in hepatocytes, inflammatory cell infiltration, and lipid degeneration in CCl4-treated group following the administration of CCl4 for 8 weeks, whereas mangiferin treatment inhibited these pathological changes, as showed by the decrease in hepatocytes degeneration, inflammatory cell accumulation and lipid deposition, indicating that mangiferin may relieve hepatic steatosis in CCl4 induced mice (Fig. 2D). Masson's trichrome staining revealed that the administration of CCl4 resulted in connective tissue proliferation, notably structure distorted and fibrous collagen deposition between the portal vein and lobules in the liver of the mice compared with the control group, suggesting that liver fibrosis was established (Fig. 2B). On the contrary, mangiferin treatment significantly decreased collagen fiber accumulation, suggesting that mangiferin has protective effect on hepatic fibrosis (Fig. 2E). In Sirius red staining, we found collagen accumulated obviously in the liver of CCl4-treated mice, and mangifeirn reversed those changes (Fig. 2C). Taken together, these data indicate that mangiferin could attenuate liver fibrosis and improve liver function in CCl4 treated mice.

### Mangiferin regulates the mRNA expression of inflammation, bile acid metabolism and fibrotic genes

**Figure 3:**
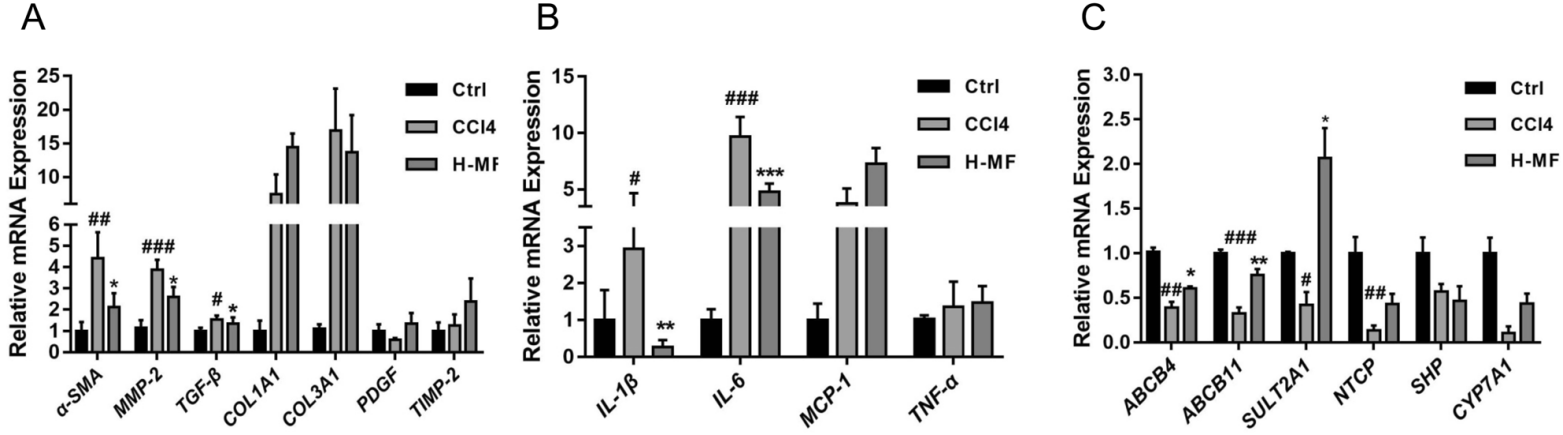
Mangiferin regulates the mRNA expression of proinflammatory cytokines, bile acid metabolism and pro-fibrotic related genes. Real time quantitative PCR were performed to detect the mRNA expression. (**A**) The mRNA expression levels of α-SMA, MMP2, TGF-β, COL1A1, COL3A1, PDGF and TIMP-2. (**B**) The mRNA expression levels of IL-1β, IL-6, MCP-1 and TNF-α. (**C**) The mRNA expression levels of ABCB4, ABCB11, SULT2A1, NTCP, SHP and CYP7A1. Date are presented as means ± S.E.M. (n = 8). #*p* < 0.05, ##*p* < 0.01 and ###*p* < 0.001, compared with control group. *p < 0.05, **p < 0.01 and ***p < 0.001, compared with CCl4 group. Ctrl, control group; CCl4, carbon tetrachloride treated group; L-MF, low dose of mangiferin (50 mg/kg); H-MF, high dose of mangiferin (100 mg/kg).

Mangiferin could play anti-fibrosis roles through NF-κB, Smad/TGF-β signaling pathways, which is related to the anti-inflammatory and anti-fibrotic properties^26^. To investigate the molecular mechanisms regarding the mangiferin protecting against CCl4 induced liver fibrosis, we measured the mRNA expression of proinflammatory cytokines, bile acid metabolism and profibrotic related genes by quantitative RT-PCR. As shown in Fig. 3A, the expression of α-SMA, a marker of hepatic stem cell activation, MMP-2 and TGF-β, the fibrogenesis related genes, were increased in CCl4-treated group, whereas these genes were significantly decreased with the treatment of mangiferin. In contrast, there was no significant different in the mRNA expression of COL1A1, COL3A1, PDGF and TIMP-2. The data indicated that mangiferin may inhibit CCl4 induced fibrogenesis via the suppression of the expression of α-SMA, MMP-2 and TGF-β in the liver. The expression of proinflammatory cytokines IL-6 and IL-1β were increased in the liver of CCl4-treated mice, while these were decreased by mangiferin treatment, however, the expression level of TNF-α and MCP-1 were not significantly changed between the groups, indicating that mangiferin may inhibit inflammatory cytokines IL-6 and IL-1β induced by CCl4 (Fig. 3B). CCl4-treatment inhibited the mRNA level of ABCB4, ABCB11, SULT2A1, NTCP and CY7A1, the bile acid metabolism related genes, in the liver of the mice. Compared with that in the CCl4-treated mice, the expression of ABCB4, ABCB11, SULT2A1 was increased in mangiferin treated group (Fig. 3C), although NTCP and CY7A1 remained unchanged.  The data indicate that the suppression of the expression of bile acid metabolism and proinflammatory related genes may also involve in the liver protective effects of mangiferin. These findings collectively demonstrated that mangiferin may relieve liver fibrosis through regulating proinflammatory cytokines, bile acid metabolism and pro-fibrotic related pathways.

### Mangiferin reduces collagen accumulation, HSCs activation and inhibits NF-κB signaling

**Figure 4:**
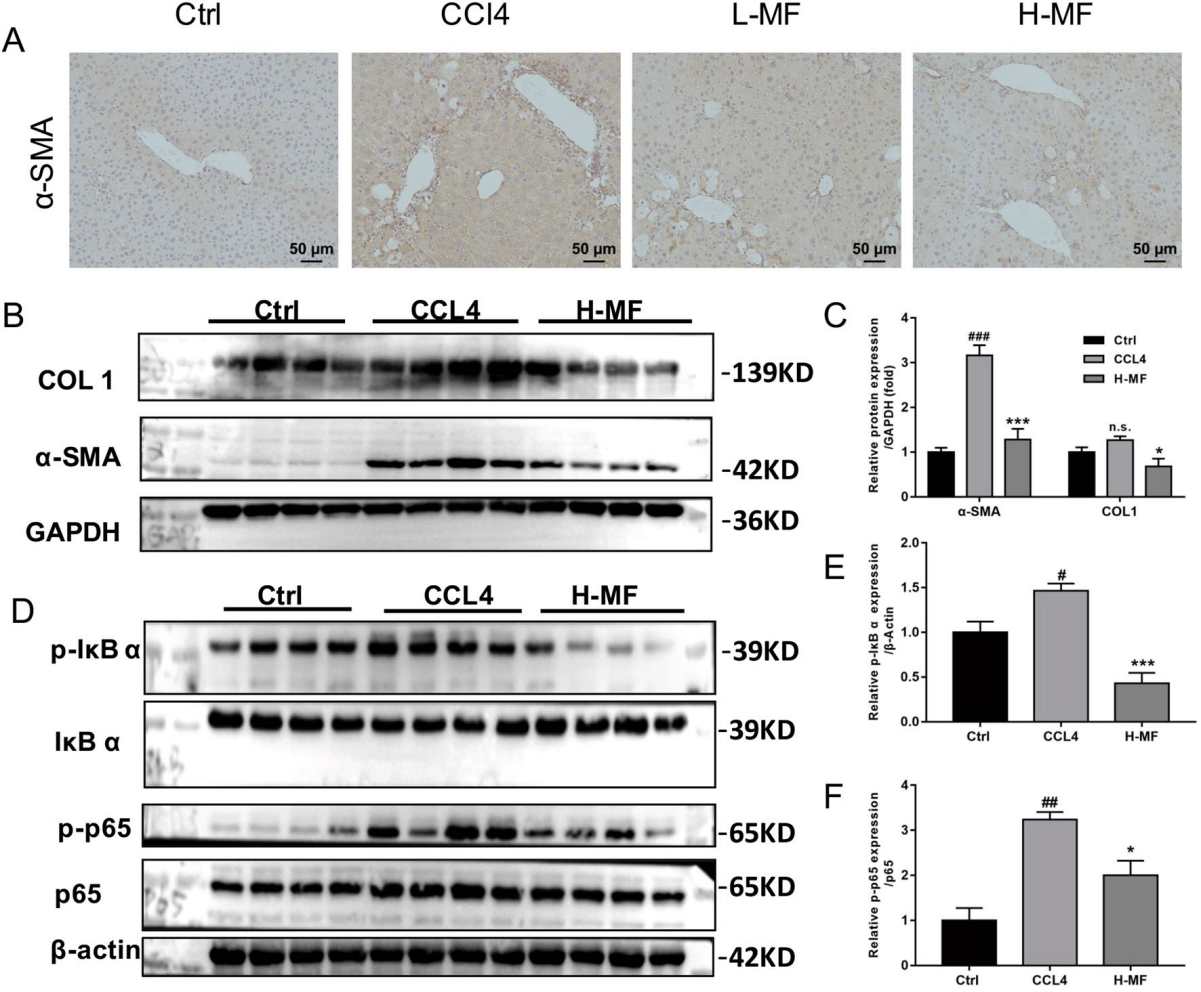
Mangiferin reduces CCl4-evoked hepatic α-SMA and COL1 protein levels and inhibits NF-κB pathway. (**A**) Immunohistochemistry staining of α-SMA. (**B**) Western blot analysis of α-SMA and COL1 protein. (**C**) Quantitation of western blot analysis of α-SMA and COL1 protein levels in (**B**). (**D**) Western blot analysis of p-IκB and p-p65 protein. (**E**) Quantitation of western blot analysis of p-IκB protein. (**F**) Quantitation of western blot analysis of p-p65 protein. GAPDH and β-Actin were assayed as an internal control respectively. All experiments repeated 3 times. The blots were cut prior to hybridisation with antibodies during blotting. #*p* < 0.05, ##*p* < 0.01 and ###*p* < 0.001, compared with control group. *p < 0.05 and ***p < 0.001, compared with CCl4 group. Ctrl, control group; CCl4, carbon tetrachloride treated group; H-MF, high dose of mangiferin (100 mg/kg).

IHC experiments showed that α-SMA levels were markedly increased in CCl4 treated mice compared to those in the control group, while mangiferin treatment repressed α-SMA levels (Fig. 4A). Next, western blot was used to verify the protein levels of COL1 and α-SMA. As shown in Fig. 4B and C, CCl4 administration upregulated COL1 and α-SMA protein expression, mangiferin decreased the protein contents of COL1 and α-SMA induced by CCl4, suggesting that mangiferin may alleviate the formation of fibrosis through the reduction of collagen accumulation and hepatic stem cell activation. Furthermore, we determined the p-IκB, p-p65 protein levels, and found that mangiferin inhibited the p-IκB protein contents (Fig. 4D,E). Meanwhile, it also inhibited p-p65 levels (Fig. 4F). Taken together, the data indicate that mangiferin may improve the liver fibrosis through inhibiting NF-κB signaling.

## Discussion

CCl4-induced liver fibrosis animals have been widely used as a model to simulate the pathogenesis of human liver fibrosis^27^. The current study demonstrated that mangiferin treatment had beneficial effects on liver fibrosis induced by CCl4. Mangiferin administration reduced CCl4-induced inflammatory cell infiltration and the release of pro-inflammatory factors, inhibited CCl4-induced NF-κB pathway activation in mice.

Overabundance of ECM deposition in the liver tissue and the activation of HSCs are the key factors involved in the formation of fibrosis and pro-fibrotic cytokines^28,29^. Previous reports have shown that mangiferin could improve renal, pulmonary, myocardial fibrosis by inhibiting the collagen deposition and the ECM aggregation in the lung, kidney and heart tissues^19–22^. In this study, we found that mangiferin reduced collagen accumulation and inhibited the mRNA and protein levels of α-SMA, the marker of the HSC activation. In addition, mangiferin also inhibited the protein level of COL1 and the mRNA levels of TGF-β, the key factor involved in the formation of fibrosis and pro-fibrotic cytokines. These data indicate that mangiferin may suppress fibrogenesis in the liver via the activation of HSCs.

Liver cell injury accounts for increasing inflammatory cell infiltration and the release of pro-inflammatory cytokines, which is a major inducer of liver fibrosis. By measuring the levels of ALT and AST, the liver injury markers, we found that mangiferin reduced CCl4-induced liver injury. The bile acid metabolism disorder usually accompanies the liver injury. Thus, we tested the expression of bile acid metabolism related genes. We found that mangiferin could reduce the bile acids accumulation in CCl4-induced mice, supporting that mangiferin may improve the liver injury induced by CCl4. Previous studies have shown that mangiferin has anti-inflammatory effects in the lung and kidney fibrosis animals^19,22^. The present study found that mangiferin alleviated inflammatory cell infiltration, and inhibited the expression of pro-inflammatory factors IL-6 and IL-1β in the liver of CCl4-induced mouse model. Considering these cytokines are the inducers of pro-fibrotic pathogenesis, we speculate that mangiferin may inhibit the hepatic fibrogenesis partly through the suppression of inflammatory signaling.

We further explored the underlying mechanism of mangiferin anti-inflammatory effect in liver fibrosis. It has been demonstrated that NF-κB is an important regulator in the series of inflammatory response and linked to the regulation of liver injury, liver fibrosis, hepatocellular carcinoma and other diseases^30^. Inhibition of NF-κB signaling could ameliorate liver fibrosis^31^. Previous studies have reported that mangiferin ameliorated renal and pulmonary inflammation by inhibiting NF-κB pathway^20,32^, and mangiferin-riched mango peel powder supplementation inhibited fibrosis and inflammatory cell infiltration in the liver of CCl_4_-induced hepatic fibrotic rats^33^. In this study, we showed that mangiferin reduced the p-IκB and p-p65 protein levels in the liver of CCl4-induced mice. Thus, the alleviating effect of mangifeirn on CCl4-induced liver fibrosis may via downregulating NF-κB signaling pathway.

## Conclusion

In conclusion, we showed that mangiferin could alleviate liver injury and inflammation, suppress the accumulation of collagen, regulate the mRNA levels of bile acid metabolism and pro-fibrotic genes in the liver of CCl4 induced mice, furthermore, mangiferin also inhibit the protein levels in NF-κB pathway. Our data suggest that mangiferin may exert anti-fibrosis effect through inhibiting NF-κB pathway and may be a potential choice for the treatment of hepatic fibrosis.

## Materials and methods

### Materials

Mangiferin was obtained from ALFA (Chengdu, China). Carbon tetrachloride (CCl4) was purchased from Aladdin (Shanghai, China).

### Animals and treatments

The procedures for this study were approved by Shanghai University of Traditional Chinese Medicine (PZSHUTCM190912014). All animal experiments in this study were conducted in accordance with the ARRIVE guidelines for reporting experiments involving animals^34^. All methods were carried out in accordance with relevant guidelines and regulations. The thirty-two male C57BL/6 mice (Six-week-old), weighing 23–29 g, were purchased from the SLAC Laboratory (Shanghai, China). All mice were housed under the controlled temperature (22–23 °C) and on a 12 h light and 12 h dark cycle with food and water ad libitum. All animals were adapted to their new housing conditions for one week before the experiments.

The mice were randomly divided into 4 groups (n = 8 in each group) as follows: control group, CCl4-treated model group, high and low dose (50 mg/kg and 100 mg/kg) of mangiferin co-treated group. Diagram of this research showed in Fig. 1A. CCl4-treated mice were injected intraperitoneally with 10% CCl4 (2 ml/kg of body weight) diluted in olive oil every other day for 8 weeks. From the 5th week, the mice were orally administered with mangiferin or same volume of vehicle for 4 weeks. At the end of the animals experiment, mice were fasted overnight. Using 20% urethane anesthetized mice and collected cardiac blood and liver tissue.

### Serum biochemical

After anesthetizing, the vascular blood was taken from the heart, and the supernatant was collected after 3000 rpm centrifugation for 15 min at the room temperature and stored at − 80 °C for further experimental analysis. The serum levels of ALT, AST, TBA, ALP and TBIL were measured by an automatic biochemical analyzer (Hitachi 7020, Japan).

### Liver histology

The liver tissues were fixed in 4% formalin solution, embedded in paraffin, cut at 5 µm and stained with haematoxylin–eosin (HE), Masson trichrome and Sirius red staining according to standard procedures. The morphology was observed with a microscope (Zeiss, Germany). The degree of fibrosis was graded by METAVIR scoring system^35^.

### Immunohistochemistry analysis

For α-SMA staining, paraffin-embedded sections were incubated with goat α-SMA polyclonal antibody (cat. no. ab5694, Lot: GR3183259-37Abcam, Cambridge, MA) overnight at 4 °C after antigen removal using sodium citrate repair solution. All slides were then incubated with goat anti-rabbit HRP secondary antibody (mp-7,452, Vector, Burlingame, CA) for 30 min at room temperature. Slides were incubated with DAB for 2 min, counterstained with hematoxylin for 2 min, and counterstained with blue reagent for 10 s.

### RNA extraction and RT-qPCR analysis

Real-time quantitative PCR was performed as previously described^36^. Briefly, Total RNA was extracted from the liver tissues using the Trizol reagent (Vazyme, Nanjing, China) according to the manufacturer's instructions. RT-qPCR was first performed using a cDNA kit (Vazyme, Nanjing, China) with 1 μg of total RNA as the template to synthesize cDNA under the following conditions: 42 °C for 2 min, 50 °C for 15 min, and 85 °C for 5 s. Quantitative real-time PCR was carried out using ChamQ Universal SYBR qPCR Master mix (Vazyme, Nanjing, China) on an ABI StepOne Plus real-time PCR system (Applied Biosystems, USA). β-actin was used as the internal reference for the expression level of mRNA of all genes. Statistical analysis was carried out by using 2^−∆∆Ct^ method. The sequences of all primers were listed in Table 1.

**Table 1:** List of primers in PCR amplification.

Gene	Forward primer	Reverse primer
β-Actin	TGTCCACCTTCCAGCAGATGT	AGCTCAGTAACAGTCCGCCTAGA
ABCB4	CGGCGACTTTGAACTAGGCA	CAGAGTATCGAACAGTGTCAAC
ABCB11	CGGACCTGTATTGTCATTGC	CCCTTCTGGTCCATCAGTTT
COL1A1	CAAGGTCACGGTCACGAA	TGGCAAAGACGGACTCAA
COL3A1	GTAGTCTCATTGCCTTGC	TCCAGAACATTACATACC
CYP7A1	GTGGTAGTGAGCTGTTGCATATGG	CACAGCCCAGGTATGGAATCA
IL-1β	TCGTGCTGTCGGACCCATAT	GGTTCTCCTTGTACAAAGCTCATG
IL-6	AACCACGGGCTTCCCTACTT	TCTGTTGGGAGTGGTATCCTCTGT
MCP-1	AGGTCCCTGTCATGCTTC	GTGCTTGAGGTGGTTGTG
MMP2	CTGTCCGCCAAATAAACC	CCCCGATGCTGATACTGA
NTCP	TATCAGCCCCCTTCAATTTC	GTGAGCCTTGATCTTGCTGA
PDGF	TTCCTGTCTCCTCCTCCC	TAACACCAGCAGCGTCAA
SHP	GGAGTCTTTCTGGAGCCTTG	ATCTGGGTTGAAGAGGATCG
α-SMA	TCGGATACTTCAGCGTCA	GGGAGTAATGGTTGGAATG
SULT2A1	GAACTGGCTGATTGAGAT	AGGTTAGAGTCGTGGTC
TGF-β	GGGAGTAATGGTTGGAATG	GGGAGTAATGGTTGGAATG
TIMP2	TGACCCAGTCCATCCAGAG	CACGCTTAGCATCACCCA
TNF-α	ATGGATCTCAAAGACAACCAACTAG	ACGGCAGAGAGGAGGTTGACTT

### Protein extraction and Western Blot analysis

The protein was extracted from the liver tissues using RIPA buffer (Beyotime, Shanghai, China) containing protease inhibitor, phosphatase inhibitor and Phenylmethylsulfonyl fluoride (PMSF). 30 μg proteins were separated using sodium dodecyl sulfate polyacrylamide (SDS-PAGE) gel, and transferred to polyvinylidene fluoride (PVDF) membranes. Blocked in 5% BSA for 2 h at the room temperature, and incubated in primary antibody for α-SMA (cat. no. ab5694, Lot: GR3183259-37, Abcam, Cambridge, MA), COL1 (cat. no. ab34710, Abcam, Cambridge, MA), IκB α (cat. no. 4814; Cell Signaling Technology), p-IκB α (cat. no. 2859; Cell Signaling Technology), p65 (cat. no. 8242; Cell Signaling Technology), p-p65 (cat. no. 3033; Cell Signaling Technology), GAPDH and β-Actin (Huabio, Hangzhou, China), with 1:1000 dilution in 3% BSA at 4 °C overnight. Then, the membranes were washed three times with TBST each 10 min, and the membranes were incubated in the secondary antibody at room temperature for 2 h. The blots were visualized using ECL chemiluminescence kit (Beyotime, Shanghai, China). GAPDH was used as loading control and Image J software (National Institutes of Health, Bethesda, MD, USA) was used for densitometric analysis of the bands.

### Statistics analysis

Statistical analyses were performed using GraphPad Prism V.7.00 (La Jolla, CA, USA). All date were presented as means ± standard error of the mean (S.E.M). Comparisons were carried out via One-way analysis of variance (ANOVA) followed by Dunnett’s tests. Differences were considered statistically significant when *p* was < 0.05.

## Supplementary Information

Supplementary Information 1.

## Data Availability

All data generated or analyzed during this study are included in this published article.

## Abbreviations

ABCB: ATP binding cassette subfamily B member
ALP: Alkaline phosphatase
ALT: Alanine aminotransferase
AST: Aspartate transaminase
a-SMA: Alpha smooth muscle actin
CCl4: Carbon tetrachloride
Col3A1: Alpha 1 chain of type III collagen
Col1A1: Collagen type I alpha 1
CYP: Cytochrome 450
CYP7A1: Cholesterol 7α-hydroxylase
ECM: Extracellular matrix
HSC: Hepatic stellate cell
IL: Interleukin
MAPK: Mitogen activated protein kinase
MCP-1: Monocyte chemoattractant protein 1
MMP: Matrix metalloproteinase
NLRP3: NOD-like receptor family: pyrin domain: containing 3
Nrf-2: Nuclear factor-E2-related factor 2
NTCP: Sodium taurocholate cotransporting polypeptide
PDGF: Platelet derived growth factor
PMSF: Phenylmethylsulfonyl fluoride
SHP: Small heterodimer partner
SULT2A1: Hydroxysteroid sulfotransferase
TBA: Total bile acid
TBIL: Total bilirubin
TGF-β: Transforming growth factor-β
TIMP-2: Tissue inhibitors of matrix metalloproteinase
TNF-α: Tumor necrosis factor-α
