# Supplementary figures and images for: Mangiferin relieves CCl4-induced liver fibrosis in mice
Source: Sci Rep. 2023 Mar 13;13:4172. doi: 10.1038/s41598-023-30582-3 (PMC10011547; doi:10.1038/s41598-023-30582-3)

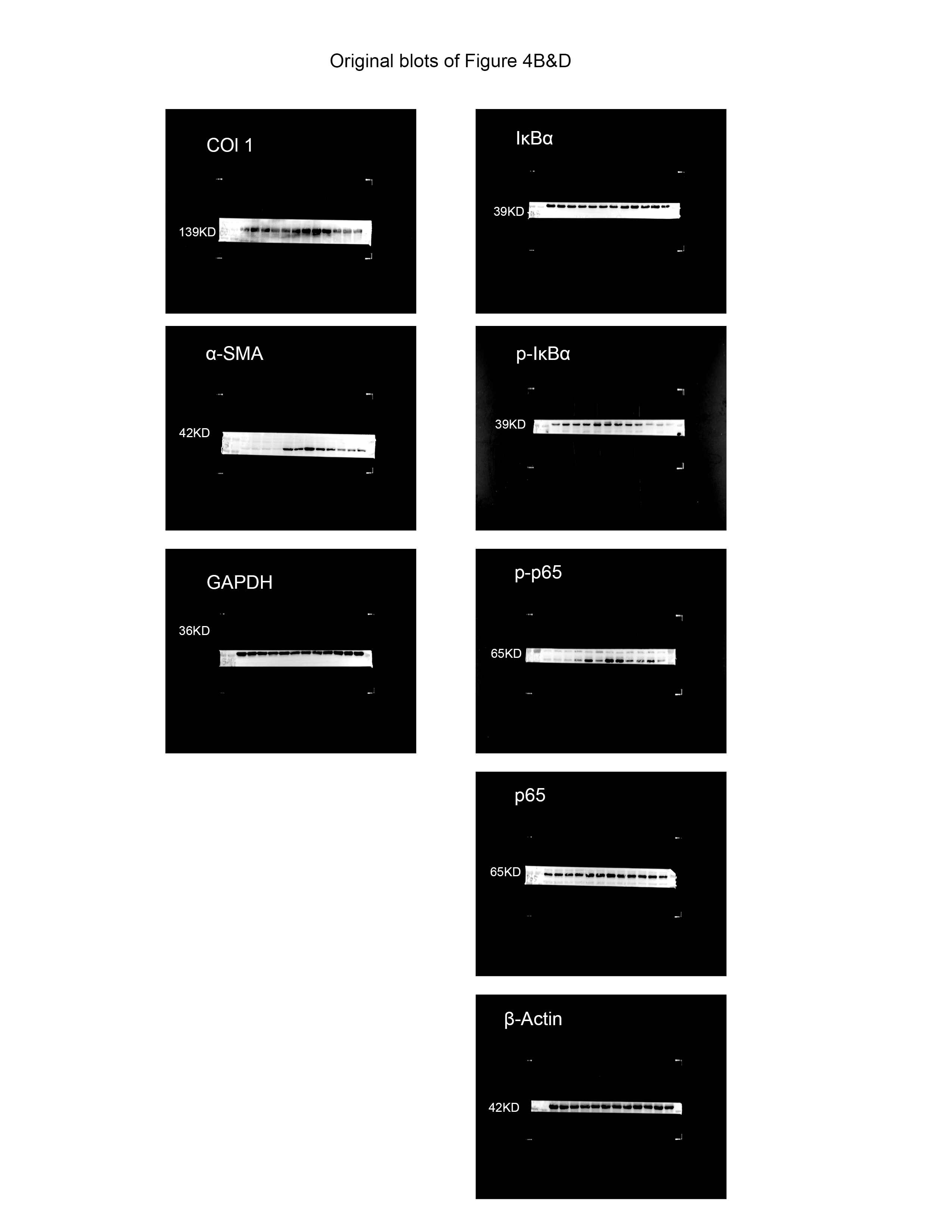

Supplement: Supplementary file 1 — Supplementary Information 1. [file 41598_2023_30582_MOESM1_ESM.jpg]
